# Innate ability of goats to sense and avoid ingestion of noxious insects while feeding

**DOI:** 10.1098/rsos.181078

**Published:** 2019-02-06

**Authors:** Tali S. Berman, Noa Messeri, Tzach A. Glasser, Moshe Inbar

**Affiliations:** 1 Department of Evolutionary and Environmental Biology, University of Haifa, Haifa 3498838, Israel; 2 Department of Biology and Environment, Faculty of Natural Sciences, University of Haifa at Oranim, Tivon 36006, Israel; 3 Ramat Hanadiv Nature Park, PO Box 325 Zikhron Ya'akov 30900, Israel

**Keywords:** noxious insects, grazing, mammalian herbivores, incidental ingestion, learning

## Abstract

Large mammalian herbivores regularly encounter noxious insects on their food plants. Recent evidence revealed that goats efficiently avoid insect ingestion while feeding, yet it is unknown whether this ability is innate. We experimentally examined the behavioural responses of naive goat kids to a common insect, the spring-webworm (*Ocnogyna loewii*). We filmed and analysed the kids' behaviour while feeding and compared it to the behaviour described in adults. Naive kids sorted the webworms apart from the food without ingesting them (all webworms survived). They exhibited behaviours similar to those displayed by adults, demonstrating an innate ability to avoid insect ingestion. The kids detected webworms using tactile stimulation, obtained by repeatedly touching the leaves with their muzzles. This enabled them to pick webworm-free leaves (leaving 93% of webworms behind). While adults frequently shook or discarded leaves with webworms or spat out webworms, these behaviours were rare in kids. The kids’ mean feeding rates doubled over the trials, indicating that their feeding efficiency on plants with and without insects improved with experience. As ingesting noxious insects could be fatal, innate avoidance is critical. These findings highlight the importance of direct interactions between mammalian and insect herbivores.

## 1. Introduction

Large mammalian herbivores (ungulates) greatly influence insect herbivores (IH) both directly and indirectly. Indirect effects of ungulates on IH, induced by changes in plant abundance, distribution, phenology, architecture and chemistry, have been thoroughly studied [1,2]. However, direct effects, such as ingestion of IH, have been largely overlooked [3].

Ungulates, which consume large quantities of diverse plants, should frequently encounter and ingest IH while feeding. Small, harmless and relatively immobile IH are ingested to some extent by ungulates [4–7]. However, ingestion of noxious IH may have adverse effects on ungulates. For example, blister beetles (Coleoptera: Meloidae) contain cantharidin that is highly toxic to ungulates [8,9]. Ingestion of setae-covered caterpillars may result in teratogenic diseases [10]. Parasites and diseases of ungulates carried by IH may be transmitted through ingestion [11,12]. Thus, the ability to avoid ingestion of noxious IH should be highly adaptive in ungulates.

Recent studies revealed that ungulates possess highly efficient behavioural mechanisms to avoid ingesting noxious IH while feeding [13,14]. Berman *et al*. [13] examined the behavioural responses of goats (*Capra hircus*) to noxious (setae-covered) spring-webworm caterpillars (*Ocnogyna loewii,*), which they often encounter while grazing in nature. They found that initial detection of the caterpillars involved tactile stimulation, obtained by repeated touching of the plant with their muzzles (probing). This behaviour enabled the goats to pick caterpillar-free plant parts to feed on. If the goats picked up a plant with caterpillars, they shook them off (by moving their head up and down), discarded the plant with the caterpillars or consumed only part of the plant (trimming). Caterpillars that entered the mouths of goats (a rare event) were spat out. As a result, 98% of the caterpillars survived and the goats were able to consume most of the food plants despite the presence of caterpillars. Currently, it is unknown whether this ability is innate or acquired through learning (individual or social).

Ungulates are born with a set of innate behaviours that influence their foraging decisions [15]. As they mature, their diet preferences are also influenced by self-experience and by observing parents and conspecifics [16–18]. The mother has the strongest influence on the diet preferences of young ungulates [19,20]. This influence can begin *in utero* [21,22] and continues after weaning, as the offspring graze with their mothers [23]. Lambs, for example, prefer food consumed by their mothers [17] and they learn to consume these foods more rapidly than lambs reared without their mothers [24]. Young ungulates may evaluate their food through trial and error. In such cases, their preferences or aversions can result from the post-ingestive effects of the food [19,25,26]. Making errors is an integral part of any learning process (social or individual), but it could be dangerous if the food item consumed is poisonous. Since inadvertently ingesting noxious IH while learning could be fatal, we hypothesized that young ungulates should possess an innate ability to avoid their ingestion.

The objective of this study was to determine whether the goats' ability to avoid ingestion of noxious IH is innate. Using dual choice and non-choice experiments, we examined the behavioural responses of naive goat kids to the presence of spring webworm caterpillars (henceforth ‘webworms’) on their food plants. Specifically, we addressed the following questions: (1) How do naive kids respond to webworms on their food plants? (2) Do they behave in a similar manner as adult goats when feeding on leaves with webworms? (3) Does the ability to avoid webworm ingestion improve with experience?

## 2. Material and methods

### 2.1. Study organisms

The study was conducted with 14 six-week-old weaned goat kids (mix of Alpine, Damascus and Mamber breeds), which were part of a herd at Ramat Hanadiv Nature Park, Israel. As the kids had never been out of the pen to forage, they did not previously encounter webworms (i.e. naive). During the experimental period, the kids stayed in a separate pen from the rest of the herd and received hay, food pellets (processed feed that includes 16% crude protein, vitamins and minerals) and water ad libitum.

Spring-webworm caterpillars are a polyphagous species common in Mediterranean-type habitats, especially in grasslands, where they can be found on a wide variety of plants, including grasses [14]. They hatch at the end of winter and the first three instars feed together inside a common web nest. Fourth and fifth instar caterpillars (which were used in the current study) disperse and feed solitarily [27]. These caterpillars are covered with long setae, which can cause skin irritations in ungulates upon contact [28]. Furthermore, ingestion of such caterpillars by ungulates may cause teratogenic diseases [10].

### 2.2. General experimental procedures

Fresh barley leaves served as food plants for both the webworms and kids. Over the week preceding the experiments, the kids received a daily portion of fresh leaves in order to become familiar with the food. The experiments were conducted in a 1 × 1 m enclosure constructed within the pen. Each trial began by leading a single kid into the enclosure to feed (voluntarily) and ended once the kid stopped feeding (left the feeding area). The kids were assigned a number and the order in which they were selected for the trials was determined using a random number table. Each kid participated only in one trial per day.

We performed dual and non-choice experiments as described by Berman *et al*. [13]. Briefly, we presented the kids with a choice between two bowls (in randomized order) containing leaves alone and leaves with webworms (henceforth ‘control leaves’ and ‘webworm leaves’, respectively). In the non-choice experiment, we presented the kids with control or webworm leaves, one bowl at a time (in randomized order). All trials, which generally lasted between 5 and 10 min, were filmed with a high definition camera (GoPro© Hero 4 black edition, GoPro Inc., San Mateo, CA, USA).

At the end of the trials, we weighed the remaining leaves and physically examined the surviving webworms to assess their state (intact or injured). We determined the kids' feeding rates as the weight of leaves consumed divided by feeding duration (g min^−1^). From the videos, we analysed their behaviour while feeding (direct observations of head and mouth movements) and examined whether it was similar to the behaviour previously described in adult goats to avoid webworm ingestion (probing, shaking, discarding, trimming and spitting, see [13]). The frequency of each behaviour was defined as the number of times it appeared per minute.

All statistical analyses were performed using IBM SPSS software v. 20 for Windows (IBM, Armonk, NY, USA). Data that did not meet the assumptions of parametric tests were tested with equivalent non-parametric tests. Specific statistical analyses for each experiment appear hereafter.

### 2.3. Analyses of the kids' responses to webworms

#### 2.3.1. Initial exposure to webworms

To study the kids' initial reactions to the presence of webworms on their food plants, we performed a dual choice experiment (as described in the ‘General experimental procedures’ section) in which we presented the kids (*N* = 14) with a choice between 3 g control leaves and 3 g leaves with three webworms. In each trial, we determined which bowl the kid began to eat from first (first bite) and which bowl the kid completely consumed first.

#### 2.3.2. Subsequent exposures to webworms

To examine whether the kids learn to avoid webworms and feed more efficiently with experience, we repeated the experiment above (with the same kids) over three consecutive days. The records of the first bite and the bowl completely consumed first were compared among trials using a sign test (Bonferroni corrected for multiple comparisons). Kids that did not finish the leaves in either of the bowls were omitted from the analysis (5/14 kids in the first trial, 4/14 in the second trial and 3/14 in the third trial). Feeding time and rates among the trials were compared using a two-way repeated measures ANOVA (with the individual kids and trials as the repeated-measures factors). As the data for the percentage of leaves consumed among the trials did not follow a normal distribution, we analysed them for each treatment (control and webworm leaves) separately using a Friedman's test. Furthermore, we compared the percentage of control and webworm leaves consumed per trial using the Wilcoxon signed-ranks test (Bonferroni corrected for multiple comparisons). For webworm leaves, we compared the frequency of behaviours that were detected among the trials using a Friedman's test. As the kids did not exhibit any behaviour while consuming control leaves in the first and second trials, no statistical analysis was performed in these cases. Finally, we compared the frequency of behaviours (that were detected) between webworm and control leaves in the third trial using a Wilcoxon signed-ranks test (Bonferroni corrected for multiple comparisons).

#### 2.3.3. Exposure to a high density of webworms

After the choice experiment ended, the kids were no longer naive to webworms. To amplify their feeding behaviour, we exposed them (*N* = 11, as not all 14 kids were cooperative) to a high density of webworms (six webworms per 3 g leaves). This was done using a non-choice experiment (as described in the ‘General experimental procedures' section), in which the kids received control leaves or high-density webworm leaves, one bowl at a time. The frequency of behaviours exhibited by the kids while consuming control and webworm leaves was compared using the Wilcoxon signed-ranks test.

## 3. Results

### 3.1. Kids’ responses to webworms on their food plants: initial and subsequent exposures

Although the kids had never encountered webworms before (naive), they were able to avoid ingesting them while feeding, without learning to do so (see the electronic supplementary material, movie S1). The kids' initial choices between webworm and control leaves (first bite) were equal across all trials (first and third trials: 7/14 chose webworm leaves; second trial: 8/14 chose webworm leaves; sign test: *p* ≥ 0.05 for all trials; Bonferroni alpha = 0.016). Most kids preferred the control leaves as they finished consuming them first throughout the trials (first trial: all nine kids completely consumed the control leaves first; second trial: 8/10 fully consumed the control leaves first; third trial: 10/11 fully consumed the control leaves first; sign test: *p* ≥ 0.05 for all trials; Bonferroni alpha = 0.016). Once they consumed the control leaves, the kids continued to feed on the webworm leaves without ingesting or harming any webworms (42/42 survived). The kids seemed to consume more control leaves than webworm leaves in all trials, yet this trend was non-significant (Wilcoxon signed-rank test: first trial Z = −2.041, *p* = 0.016; second trial Z = −1.298, *p* = 0.194; third trial Z = −2.032, *p* = 0.042; Bonferroni alpha = 0.016; table 1). In addition, the percentage of leaves consumed among the trials was similar for both control and webworm leaves (Friedman test: control leaves $\chi_{2}^{2}=0.56$, *p* = 0.756; webworm leaves $\chi_{2}^{2}=1.824$, *p* = 0.402; Bonferroni alpha = 0.016; table 1).

**Table 1.** The effect of webworms on the percentage of leaves consumed and the amount of time spent feeding by kids (dual-choice trial). The mean percentage of leaves consumed and mean feeding time were similar between control and webworm leaves across all trials (non-significant). *N* = 14 kids. Means ± s.e.

trials	mean percentage of leaves consumed (%)		mean feeding time (s)	
	without webworms	with webworms	without webworms	With webworms
1	81 ± 9	58 ± 9	55.5 ± 9.1	60.5 ± 9.3
2	83 ± 8	76 ± 9	34.5 ± 4.0	40.9 ± 5.5
3	93 ± 5	74 ± 11	34.7 ± 3.8	26.3 ± 4.2

Feeding time was alike between control and webworm leaves (two-way repeated measures ANOVA, *F*_1,13_ = 0.066, *p* = 0.802; table 1), yet it halved between the first and third trials for both treatments (two-way repeated measures ANOVA, *F*_2,26_ = 7.56, *p* = 0.013; table 1). The interaction between the treatment (control versus webworm leaves) and the trials was non-significant (two-way repeated measures ANOVA, *F*_1,13_ = 1.064, *p* = 0.360).

**Figure 1.**
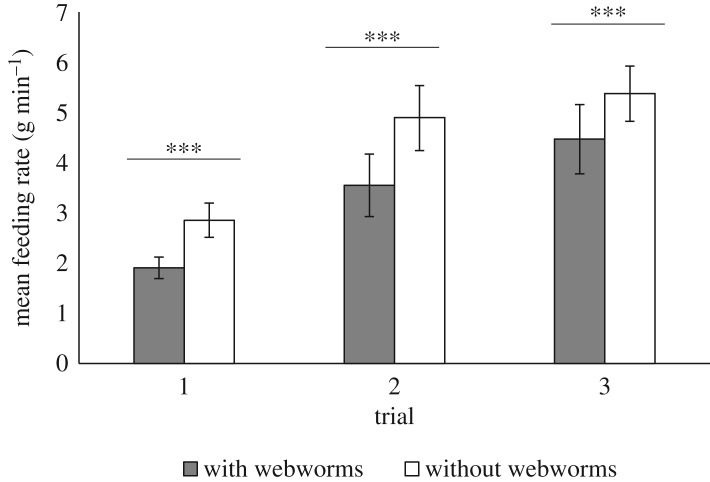
The effect of webworm presence on the kids' feeding rate over three consecutive dual-choice trials. *N* = 14 kids. Bars show means ± s.e. ***p ≤ 0.001.

The mean feeding rate was 1.5 times lower when webworms were present on the leaves (two-way repeated measures ANOVA, *F*_1,13_ = 27.292, *p* < 0.001; figure 1). Nonetheless, the kids’ mean feeding rates doubled between the first and third trials, whether webworms were present or not (two-way repeated measures ANOVA, *F*_2,26_ = 6.534, *p* = 0.005, figure 1). The interaction between the treatment (control versus webworm leaves) and the trial was non-significant (two-way repeated measures ANOVA, *F*_1,13_ = 1.612, *p* = 0.219).

**Figure 2.**
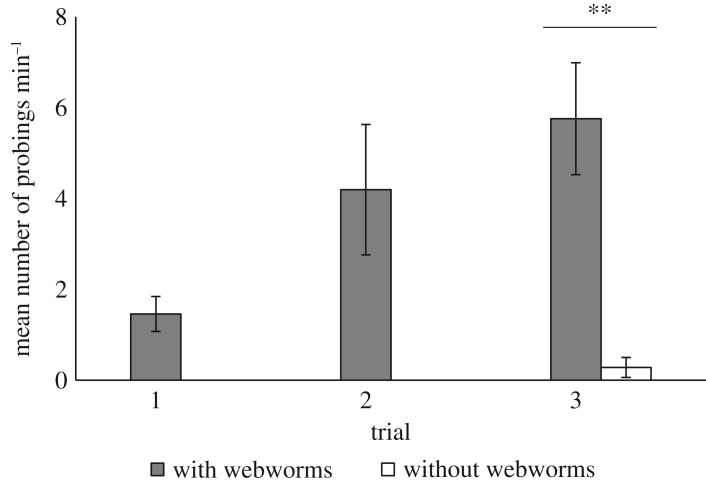
Mean number of probings min^−1^ by kids while feeding on leaves with and without webworms over three consecutive dual-choice trials. *N* = 14 kids. Bars show means ± s.e. **p ≤ 0.01.

The types of behaviours exhibited by kids to avoid webworm ingestion were similar to those displayed by adults (probing, shaking and discarding). No new behaviours were observed. The videos revealed that probing was the main behaviour that enabled the kids to avoid webworm ingestion (see electronic supplementary material, movie S2). Probing mostly occurred when webworms were present on the leaves and did not appear at all when feeding on control leaves in the first and second trial (third trial: Wilcoxon signed-rank test, *Z* = −2.746, *p* = 0.006; Bonferroni alpha = 0.025; figure 2). Interestingly, the kids exhibited probing from their first encounter with the webworms (first trial). Although the number of probings min^−1^ seemed to increase over the trials, this trend was non-significant (Friedman test, $\chi_{2}^{2}=1.849$, *p* = 0.397; Bonferroni alpha = 0.025; figure 2). Shaking and discarding were rarely observed in either of the treatments (a mean of less than one occurrence per trial for both webworm and control leaves, data not shown). Like probing, they appeared from the first encounter with the webworms. Spitting was not seen at all as webworms never entered the kids' mouths.

### 3.2. Kids’ responses to webworms on their food plants: exposure to a high density of webworms

Increasing the number of webworms (see electronic supplementary material, movie S3) on the leaves in the non-choice experiment did not affect the frequency of the different behaviours. Probing continued to be the most frequent behaviour exhibited by the kids and it was 16 times more common in webworm leaves compared to control leaves (4.11 versus 0.25 probings min^−1^, Wilcoxon signed-rank test, Z = −3.059, *p* = 0.002). Shaking and discarding were still uncommon (a mean of less than one occurrence per trial for both webworm and control leaves, data not shown) and spitting was not observed as the webworms never entered the kids' mouths.

Overall, in both the choice and non-choice experiments, probing enabled the kids to pick webworm-free leaves, leaving behind a mean of 93% webworms (184 of 198). The few webworms picked up with the leaves (7%, 14 of 198) mostly escaped (dropped off) or were shaken off or discarded by the kids.

## 4. Discussion

Recent evidence revealed that goats possess highly efficient behavioural mechanisms to detect and avoid ingesting noxious IH while feeding [13]. Here, we show that this ability is clearly innate. By using behaviours (and senses) similar to those of adults, naive kids were able to detect webworms on their food plants and successfully feed without ingesting (or harming) any of them. While the ability to avoid IH ingestion is innate, the efficiency of feeding on plants with and without IH improved with experience. As ingestion of certain IH may adversely affect ungulates [9,10,29], the ability to avoid it from birth is highly advantageous.

### 4.1. Naive kids’ responses to webworms on their food plants

Naive kids, like adults, efficiently avoided webworm ingestion. They did not avoid leaves with webworms due to their innate ability to sort them apart from the food (see electronic supplementary material, movies S1 and S3). When given a choice between control and webworm leaves, the kids preferred control leaves which were easier to handle, as predicted by the optimal foraging theory [30,31]. When given no choice (or once the kids had entirely consumed the control leaves), they fed on the webworm leaves; however, they eventually gave up on these leaves when sorting the webworms apart from the food took too long (this trend was maintained over the trials, table 1). Overall, the kids' responses to webworms were quite similar to those of adults [13]. While grazing in a competitive environment, ungulates (especially young individuals) must be able to use as much of their food source as possible despite the presence of IH.

The kids’ mean feeding rates doubled between the first and third trials, whether webworms were present or not, indicating that their feeding efficiency improved with repeated exposure and practice. Indeed, the kids' feeding durations decreased over the trials and the amount of food they consumed seemed to increase for both control and webworm leaves (although this trend was non-significant). Similarly, it has been shown that the efficiency of food selection in young ungulates improves with experience [15,32,33]. Foraging efficiency also improves with age [32,34]. This can explain why the mean feeding rate of kids was approximately 13 times lower than that of adults for both treatments [13]. In addition, adult goats have larger mouths than kids, allowing them to consume more food in a shorter time period [32,35]. While the adults’ mean feeding rates were similar between the treatments, the kids' feeding rates were significantly lower when webworms were present. The lack of experience in sorting and handling leaves with IH on them is probably accountable for this difference between the treatments.

### 4.2. Kid behaviour while feeding on plants with webworms

Ungulates are born with a set of behaviours that enable them to avoid numerous hazards, including inadequate food items [15]. Overall, the types of behaviours exhibited by kids to avoid webworm ingestion were similar to those displayed by adults, and included probing, shaking and discarding [13]. Nonetheless, the frequency of these behaviours (mostly shaking and discarding) was lower in kids. Naive kids exhibited the behaviours from their first encounter with the webworms, demonstrating a clear innate ability to avoid IH ingestion. While these behaviours efficiently prevent IH ingestion, they are probably routinely used by ungulates while selecting or avoiding specific plant parts, regardless of the presence of IH.

Probing was the main behaviour employed by kids (as by adults) when feeding on leaves with webworms, and it enabled them to pick webworm-free parts to feed on while leaving 93% of the webworms behind. The number of probings min^−1^ seemed to increase with experience (this trend was non-significant probably due to a limited number of replications, figure 2). Nevertheless, this result suggests that like feeding rate, the ability to efficiently perform probing may improve with experience.

While adults mostly relied on shaking to dislodge webworms that were picked up with the leaves, this behaviour was infrequent in kids (even with higher numbers of webworms). As the kids fed relatively slowly (approx. 13 times slower than adults), they might have been able to better choose leaves without webworms on them. Furthermore, slow handling of the food may have enabled the mobile webworms to escape the leaves as they were picked up by the kids (reducing the need for shaking them off). Indeed, certain IH, including caterpillars, are known to escape their plant when in danger, in order to prevent injury or death [7,36–39].

While webworms occasionally entered the mouths of adults, they never entered the mouths of kids. Adult goats have larger mouths than kids, allowing them to take bigger bites, ingest more food and feed faster [32,35]. Hence, webworms are more likely to accidentally enter the adults’ mouths while feeding [13]. Nonetheless, spitting is probably innate (like the other behaviours) and should aid kids in ejecting smaller IH that may enter their mouths. As the kids learn to feed faster, the need to perform dislodging behaviours, such as shaking and spitting, is likely to increase.

### 4.3. How kids detect webworms on their food plants

Ungulates select and discriminate between food items using sight, smell, touch and taste [40,41]. Kids, like adults, detected the webworms on their food only after their muzzle directly contacted the leaves (touch), demonstrating that sight and smell are probably not involved in this initial process. If sight or smell were important in detecting webworms, the kids would have probably started eating (first bite) from the control leaves first after becoming familiar with the webworms (during the first trial). Yet their initial choice in all trials was random.

The prevalent probing behaviour employed by kids feeding on leaves with webworms indicates that, like adults, kids rely on touch to detect webworms. Goats have sensitive and mobile lips that aid them in examining and picking food items [42–44]. By touching the leaves with their muzzles, kids were able to accurately detect the webworms on the leaves and avoid their ingestion. Adults also relied on taste, in addition to touch, to detect webworms. Taste is highly important in ungulates because it is the last sense used to evaluate food properties before ingestion [41]. Therefore, taste (combined with touch) is also likely to enable kids to detect IH that may enter their mouths.

Using touch to detect IH is advantageous to ungulates as it is effective during day and night, not influenced by odours, visibility or the location of the IH on the plant. Despite their ubiquity on plants, IH may not always stand out visually and chemically (odour) [45–47]. Hence, the ability to accurately detect IH on the plant, regardless of the environmental conditions, is critical to the ungulates.

## 5. Conclusion

When a kid approaches leaves with webworms, it does not completely avoid feeding on them, rather, it innately uses its senses and efficient behaviours (mainly probing) to avoid webworm ingestion while feeding. Young ungulates mostly learn which foods to eat and which to avoid through individual and social (mother or conspecifics) learning [16–18]. While learning is important in shaping the young ungulate's diet, it is quite clear why they are born with behaviours that enable them to avoid noxious IH ingestion. Nonetheless, their ability to use plant matter with IH on it improves with practice and experience.

Direct trophic interactions between mammalian herbivores and IH have been largely overlooked [3]. Yet, recent evidence shows that ungulates possess efficient behavioural mechanisms to avoid IH ingestion [13,14]. The current study demonstrates that these behavioural mechanisms are innate. The fact that ungulates are born with the ability to avoid IH ingestion further highlights that these interactions are prevalent and important. Encounters between ungulates and IH in nature are inevitable. By employing effective behaviours, ungulates are able to prevent direct consumptive interactions throughout their life, benefiting both themselves and IH as they feed together in the same habitat.

## Supplementary Material

Supplementary movies 1 - 3

## Data accessibility

Data are available from the Dryad Digital Repository: http://dx.doi.org/10.5061/dryad.v3539d2 [48].
