# Supplementary material for: Innate ability of goats to sense and avoid ingestion of noxious insects while feeding
Source: R Soc Open Sci. 2019 Feb 6;6(2):181078. doi: 10.1098/rsos.181078 (PMC6408377; doi:10.1098/rsos.181078)
Supplement: Supplementary movies 1 - 3 [file rsos181078supp1.docx]

**Innate ability of goats to sense and avoid ingestion of noxious insects while feeding**

Tali S. Berman^a^, [talisberman@gmail.com](mailto:talisberman@gmail.com)

Noa Messeri^a,b^, [noamesseri1@gmail.com](mailto:noamesseri1@gmail.com)

Tzach A. Glasser^c^, [Tzach@ramathanadiv.org.il](mailto:Tzach@ramathanadiv.org.il)

*Moshe Inbar^a^, [minbar@research.haifa.ac.il](mailto:minbar@research.haifa.ac.il)

^a^Department of Evolutionary and Environmental Biology, University of Haifa, Haifa, 3498838, Israel

^b^Department of Biology and Environment, Faculty of Natural Sciences, University of Haifa at Oranim, Tivon, 36006, Israel

^c^Ramat Hanadiv Nature Park. POB 325 Zikhron Ya'akov, 30900, Israel

*Corresponding Author**:** Moshe Inbar, Department of Evolutionary and Environmental Biology, University of Haifa, Haifa 3498838, Israel, phone number: + 972 4 8288767, e-mail: [minbar@research.haifa.ac.il](mailto:minbar@research.haifa.ac.il)

Keywords: noxious insects, grazing, mammalian herbivores, incidental ingestion, learning

**Video captions and legends**

**
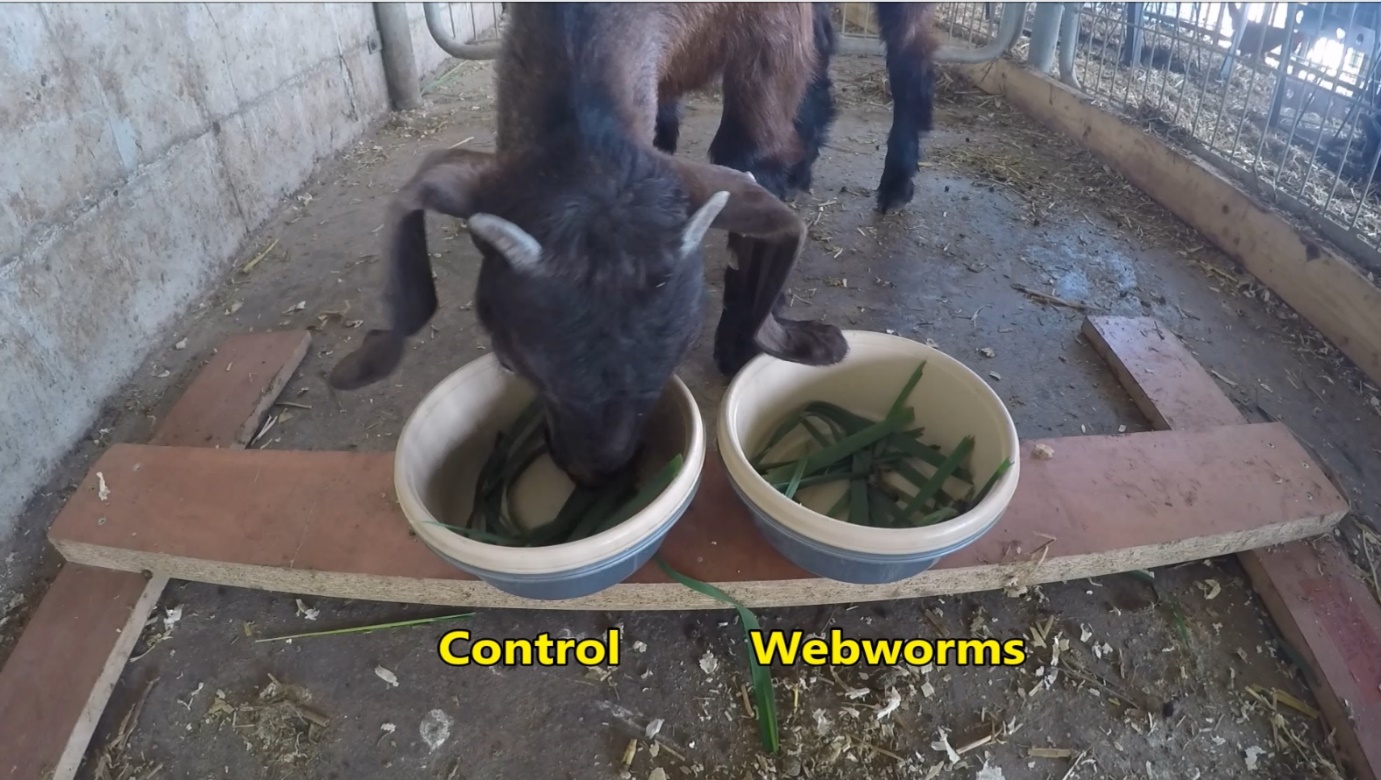
**

**Supplementary movie 1.** A naive kid presented with a choice between control (left bowl) and webworm leaves (right bowl). The kid began feeding from the webworm leaves (first bite) yet, proceeded to feed from the control leaves. Once the control leaves were completely consumed, the kid fed on the webworm leaves while efficiently avoiding webworm ingestion. Video speed increased by ×5.

**
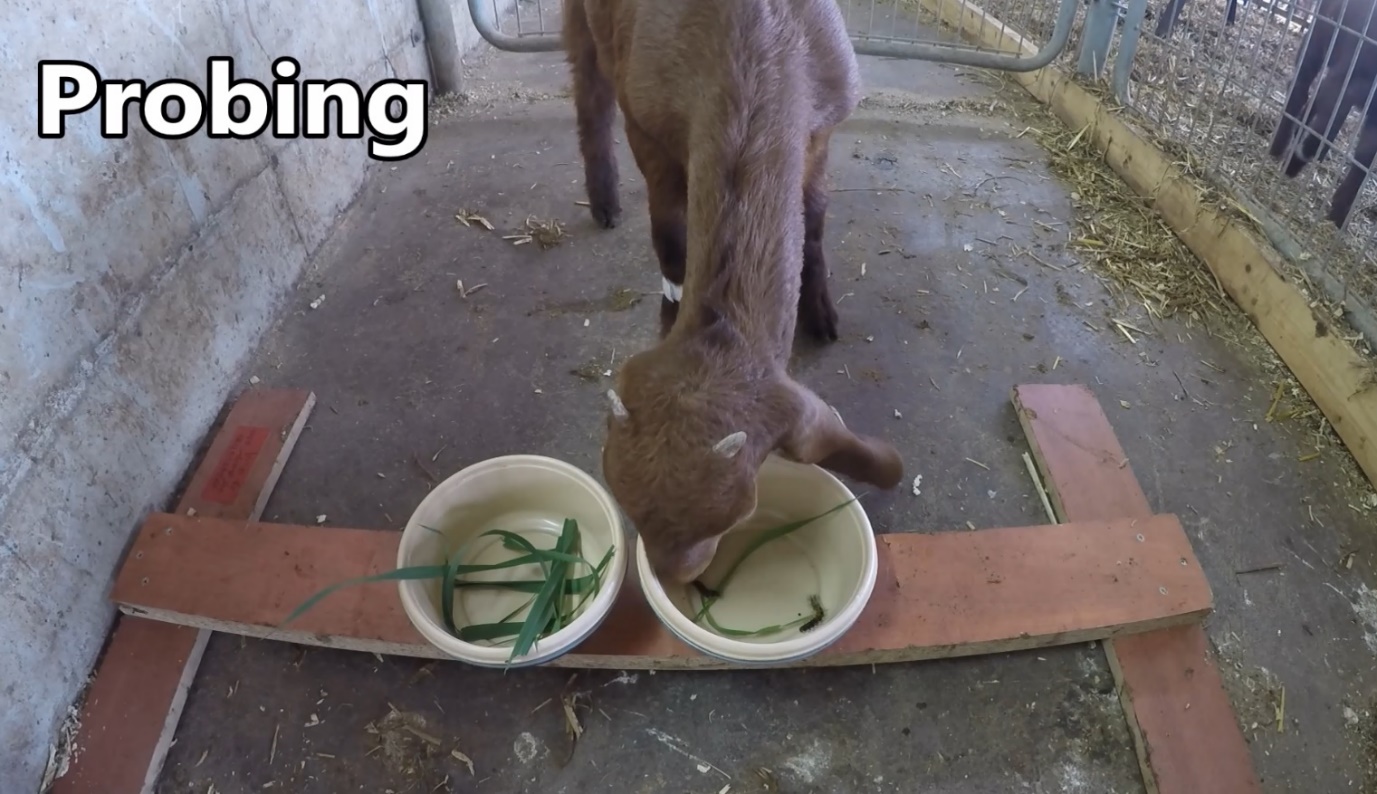
**

**Supplementary movie 2.** Probing behaviour while feeding on leaves with webworms (right bowl). By repeatedly touching the leaves with its muzzle (probing), the kid was able to pick webworm-free leaves. Video speed increased by ×5.

**
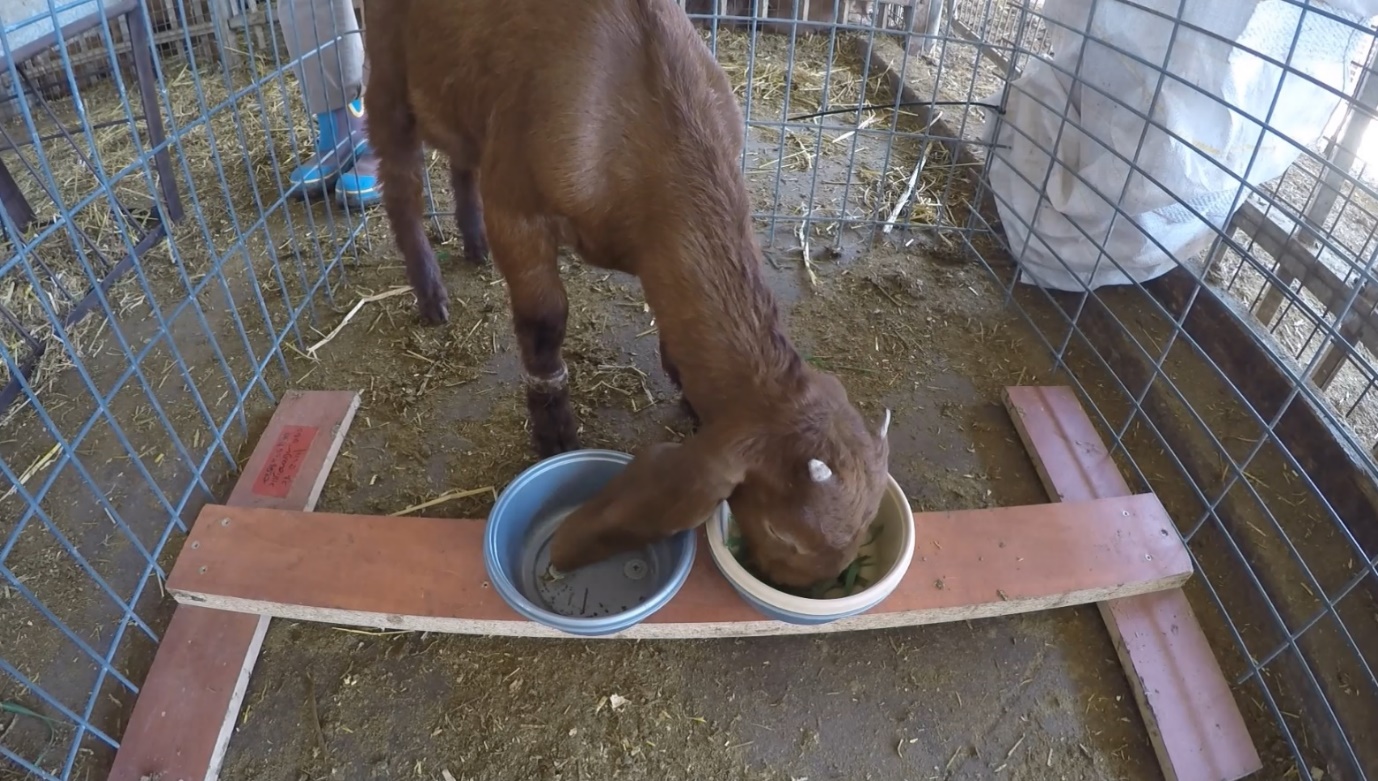
**

**Supplementary movie 3.** A feeding kid (not naïve) avoiding the ingestion of numerous webworms (non-choice trial). All six webworms remain intact. Video speed increased by ×5.
